# The Rate of Torque Development as a Determinant of the Torque–Velocity Relationship

**DOI:** 10.1111/sms.70035

**Published:** 2025-03-14

**Authors:** Gennaro Boccia, Paolo Riccardo Brustio, Francesco Salvaggio, Ludovico Grossio, Elena Calcagno, Arianna Pintore, Alberto Rainoldi, Pierre Samozino

**Affiliations:** ^1^ Department of Clinical and Biological Sciences University of Turin Torino Italy; ^2^ Neuromuscular Function Research Group, School of Exercise and Sport Science University of Turin Torino Italy; ^3^ Department of Neuroscience, Biomedicine and Movement Sciences University of Verona Verona Italy; ^4^ Department of Medical Sciences University of Turin Torino Italy; ^5^ Univ Savoie Mont Blanc Interuniversity Laboratory of Human Movement Sciences Chambéry EA France

**Keywords:** explosive contractions, force–velocity relationship, HD‐EMG, knee extensors, maximal power

## Abstract

We investigate the contribution of isometric rate of torque development (RTD) and maximal voluntary torque (MVT) to the dynamic force production capacities of knee extensors obtained from the torque–velocity (TV) relationship, that is, the theoretical maximal velocity (*V*_0_), torque (*T*_0_), and maximal power (*P*_max_). Single‐leg knee extensors were tested in 64 young adults (31 females). RTD and root mean square (RMS) of electromyographic signals from the knee extensors were recorded during isometric and incremental load dynamic (nonisokinetic) contractions. In the dynamic test, torque and velocity were continuously measured and averaged over 80°–140° knee angles to determine individual TV relationships. TV relationships were well fitted by hyperbolic regression (*r* ^2^ from 0.983 to 0.993). Stepwise linear regressions showed that the main determinant of *V*_0_ was normalized RTD50 (*R* ^2^ = 0.145, *p* = 0.004); the main determinant of *T*_0_ was MVT (*R* ^2^ = 0.760, *p* < 0.001); and the main determinant of *P*_max_ was RTD150 (*R* ^2^ = 0.612, *p* < 0.001). *V*_0_ (when obtained from averaged values over knee extension) is partially explained by rapid torque capacity (i.e., “explosive strength”). Therefore, the capacity to produce torque at high velocity partly depends on the capacity to rise quickly the torque in the early phase of the contraction, suggesting that some underlying determinants of RFD would also affect *V*_0_.

## 1 Introduction

High‐intensity ballistic performances in sport activities (e.g., jumping, throwing, sprinting) or success in some daily life tasks for fragile people (e.g., chair lift, stair climbing) depend directly on the mechanical impulse applied to the body or to the external mass to move. From dynamics principles, the higher the averaged external torque over the range of motion, the higher the velocity at the end of the movement, and the higher the performance or the success in the task. However, dynamic torque production capacities of the neuromuscular system decrease when the velocity increases [1]. This has been well described by torque–velocity (TV) and power–velocity (PV) relationships, widely studied during single‐joint (e.g., knee extension [2, 3]) or multijoint (pedaling, jumping, leg press, sprinting [1, 4, 5]) movements. These relationships have been characterized by their extrema: the maximal theoretical torque at null velocity (*T*_0_), the maximal theoretical velocity until which torque can be produced (*V*_0_), and the maximal power output (*P*_max_, apex of the PV curve). While the general shape of TV and PV relationships is the same for everybody (curvilinear and linear for single‐joint and multijoint tasks [1, 3]), high interindividual variabilities have been reported for *T*_0_, *V*_0_, and *P*_max_ variables across genders, ages, sport activities, athlete's position, or training history [6, 7, 8]. If peak values of torque or power and associated velocity were used in some cases to draw these relationships [9, 10], averaged values over the entire movement are more interesting when focused on torque production capacities and functional performances [4, 5, 11, 12]. However, at each time of the movement, the maximal external torque the neuromuscular system can produce depends on the muscle–tendon unit shortening velocity, length, and the time from the beginning of the contraction [13, 14]. So, whatever the velocity, for a given task over a given range of motion, the maximal averaged torque is affected by the rate of torque development at the very beginning of the contraction.

During knee extension, the rate of torque development (RTD) is the ability to rapidly increase muscle torque following the initiation of a rapid contraction [15, 16]. While assessed during isometric contraction, a high RTD of the knee extensors plays a key role in various explosive activities such as vertical jumping [17], weightlifting, [18] and cycling [19]. In addition, a high RTD has been shown to be beneficial in tasks such as endurance running and everyday functional movements [20, 21]. These activities are characterized by short burst‐like contractions, usually performed at high velocities and typically lasting less than 200 ms [22]. The variability among individuals in the RTD of knee extensors can be partially attributed to variations in maximal voluntary torque (MVT). However, MVT seems to exert a more significant influence in the later phase of the contraction, emerging as the primary determinant of RFD from 75 ms onward [23, 24, 25]. This mostly explains the correlation reported between the later phase of the RTD (≥ 100 ms) and quadriceps muscle thickness [24] or volume [26]. The contribution of neural and contractile mechanisms varies throughout the time course of the torque–time curve rise. In isometric contractions, the amplitude of agonist muscle activation, as assessed through electromyography (EMG) within the initial 50 ms from contraction onset, demonstrates a strong correlation (*r* ≈ 0.7–0.8) with RTD during the first 50 ms [23, 24, 25, 27, 28, 29]. The elevated EMG amplitude during the initial 50 ms of ballistic contractions likely reflects the compressed motor unit recruitment and high firing rates [30]. However, the relationships between (i) early muscle activation and early RTD, assessed during isometric contraction, and (ii) dynamic torque production capacities during nonisokinetic movement, characterized by TV and PV relationships, have not been tested yet.

The aim of this study was thus to test the effect of RTD, and of the underlying early muscle activation, on the interindividual variabilities in TV and PV relationships of knee extensors. Whatever the velocity, a low RTD was supposed to decrease the averaged torque produced over the knee extension due to a lower torque in the first 100 or 200 ms of the contraction. The averaged torque alteration was thus expected to be more pronounced during short and fast movements than during long and slow ones. Consequently, the interindividual variabilities in isometric RTD and early muscle activation (< 50 ms) were supposed to explain variabilities in dynamic torque production capacities at high (*V*_0_), but not at low (*T*_0_) velocities, and so in maximal power (*P*_max_).

## 2 Material and Methods

### 2.1 Participants

Sixty‐four young and healthy subjects, of which 31 were females (mean ± SD = 23 ± 2 years; 166.3 ± 5.6 cm; 59.8 ± 7.9 kg) and 33 were males (25 ± 4 years; 176.1 ± 6.4 cm; 71.2 ± 9.6 kg), were included in the study. Participants were physically active, practicing leisure physical activity at least 2 times per week. Exclusion criteria included any previous history of neuromuscular disorders or lower limb injury in the previous 6 months. All the participants were informed about the testing procedure and provided written informed consent prior to their participation in this study, which was approved by the Ethical Advisory Committee (University of Torino—approval no: 0432975 of July 21, 2023) and performed in accordance with the Helsinki Declaration. Participants visited the laboratory only once and avoided strenuous exercise for 24 h before the experimental session.

The experimental session was divided into three parts: (1) warm‐up, (2) familiarization and testing on the isometric ergometer, (3) familiarization and testing on the dynamic nonisokinetic ergometer. All measurements were taken from the participants' right lower limbs (which was the dominant limb in 55 of 64 participants).

### 2.2 Experimental Setup

**FIGURE 1:**
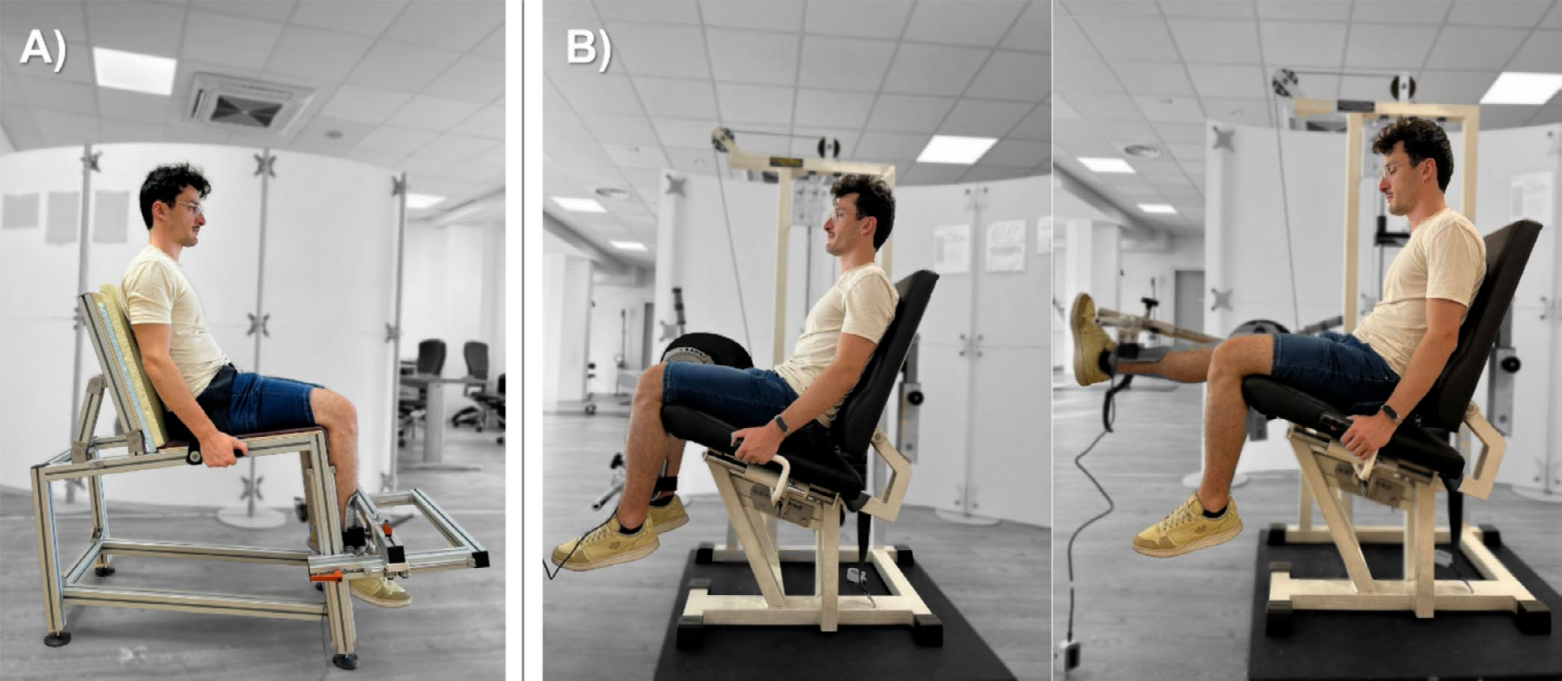
Experimental setup of the (A) isometric and (B) dynamic task. For the dynamic task, a modified commercial leg extension machine was instrumented with a linear encoder to detect the position of the weight stack and a strain gauge and ship protector were placed on the arm of the machine and positioned in the same place as the isometric testing.

For both isometric and dynamic testing, the participants’ knee and hip were flexed at 80° from full extension at the starting position. For the isometric testing, the participants were seated on a custom‐made chair that allowed the assessment of the right knee extensors (Figure 1A). Straps were fastened across the chest and hips to avoid lateral and frontal displacements. The strain gauge load cell (546QD—220 kg; DSEurope, Milan, Italy) was positioned 2 cm above the malleolus, perpendicular to the tibial alignment. To avoid pain and maintain structural stiffness, a standard hard shin protector was placed between the thrust surface and the tibia. For the dynamic testing, a modified commercial leg extension machine (leg extension R.O.M., Technogym, Cesena, Italy) was instrumented with a linear encoder (DWT01010KCN1; MAFtec, Milan, Italy) to detect the position of the weight stack (Figure 1B). Furthermore, a strain gauge and ship protector were placed on the arm of the machine and positioned in the same place as in the isometric testing.

### 2.3 Procedures

First, the skin of the participants was prepared for electrode locations (see the High‐Density Surface Electromyography section next). Then participants performed 5 min of pedaling at 90 W. Afterward they were seated on the isometric testing ergometer. The protocol started with a warm‐up comprising 10 submaximal isometric contractions ranging from 20% to 80% of the perceived maximum torque and the familiarization with ballistic contractions until they were able to produce proper burst‐like contractions (details next).

To measure MVT, two 5‐s maximal voluntary contractions were performed with 2 min of rest in between. Participants were instructed to push as hard as possible for 5 s, and they received standardized strong verbal encouragement. Participants also received visual real‐time feedback regarding the torque response through a screen placed in their line of sight.

**FIGURE 2:**
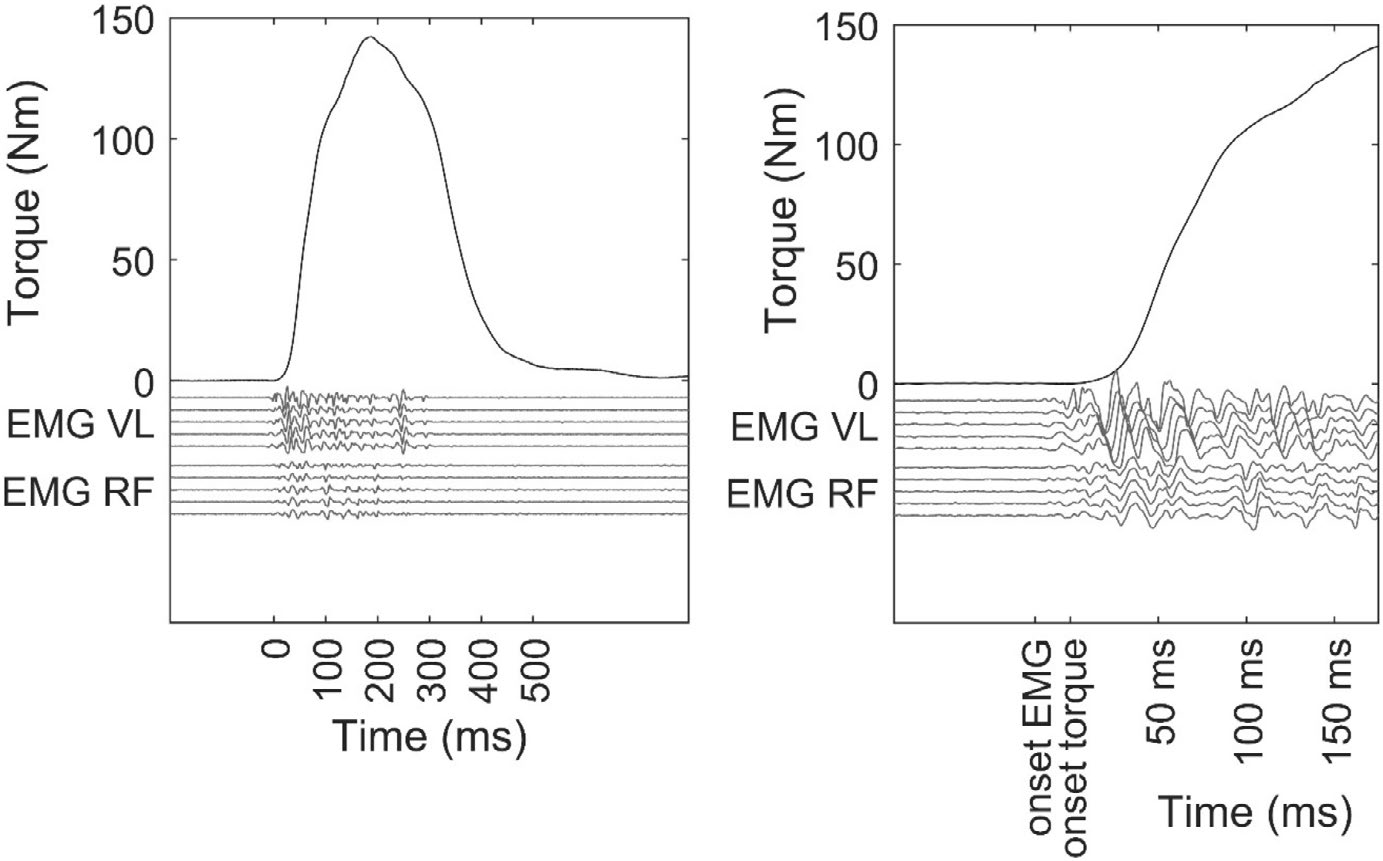
Torque and high‐density electromyographic (HD‐EMG) signal recorded during a burst‐like rapid contraction are shown for a representative participant. The HD‐EMG signal is shown for a column of electrodes from the vastus lateralis (VL) and rectus femoris (RF) muscles. The left panel shows that the active phase was about 200 ms long. The right panel, which is a magnification of the left panel, shows the onset of torque and the 50, 100, 150 ms time intervals from torque onset. The EMG onset was arbitrarily set at 20 ms before the torque onset in accordance with previous studies of the quadriceps in voluntary contraction. The time intervals for the EMG were then advanced (shifted to the left) by 20 ms compared to those for the torque.

After 2 min of rest, the participants performed 10 explosive contractions (brief pulses) interspersed by 15 s of rest (Figure 2). The contractions were characterized by a short active phase (lasting ≈200 ms) and by avoiding any holding phase, resulting in a burst‐like shape (Figure 2, left panel). Participants were instructed to push “as fast and as hard as possible” [31]. A visual line on the screen depicted 70% of MVT during the contractions, and participants were instructed to achieve a peak torque above this level during each explosive contraction [23].

**FIGURE 3:**
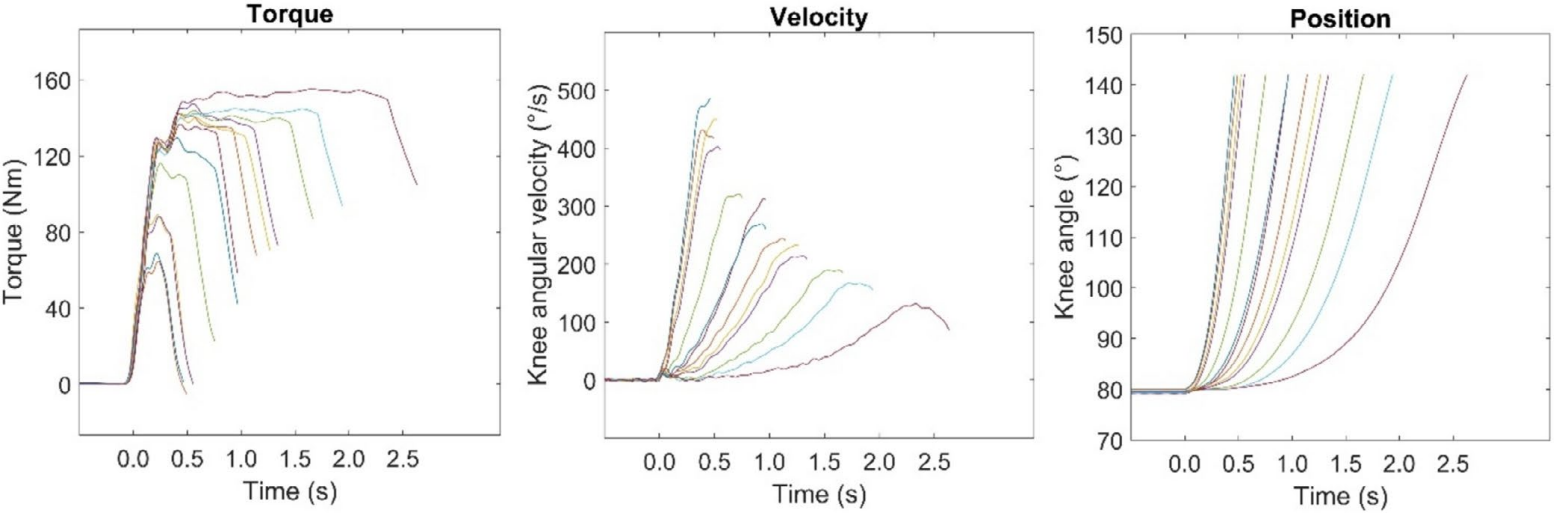
The torque, velocity, and position signals recorded during the incremental load test at the dynamic nonisokinetic leg extension are shown for a representative participant. The analysis was bounded from 80° (starting angle) to 140° of knee extension (right panel) because this range of motion was covered by all participants in every load, without any active deceleration at the end of the contraction. The torque was calculated from the force measured by the strain gauge load cell positioned above the tibia, while the angular displacement was determined using the linear encoder attached to the weight stack.

After 2 min of rest, participants moved to the non‐isokinetic leg extension machine for the incremental load protocol. Participants were familiarized with the dynamic task with at least 10 contractions carried out with low and intermediate loads (depending on individual self‐perception). They were instructed to push as fast and as hard as possible and to continue to accelerate with maximal effort throughout the entire range of motion (i.e., from ≈80° to ≈140° of knee extension). For the testing, the loads were presented in ascending order, starting with three standardized loads for each subject (i.e., 2.5, 7.5, and 15 kg). This was followed by at least four additional incremental loads, up to the load that the participant was unable to move despite maximal effort. Participants performed two attempts with the first three loads (2.5, 5, and 15 kg) and only one attempt for the further loads. The rest interval between two trials with the same load was 30 s, while the interval between two different loads was 2 min. The operator should select the loads and try to distribute them evenly to cover the entire load range from the lightest to the heaviest. Representative signals recorded during the incremental load protocol are reported in Figure 3.

#### 2.3.1 High‐Density Surface Electromyography

High‐density surface electromyography (HDsEMG) signals were recorded from the vastus lateralis (VL) and rectus femoris (RF) muscles in monopolar configuration using two matrices of 64 electrodes each (13 rows × 5 columns, 8 mm interelectrode distance, gold‐coated; model: GR08MM1305, OT Bioelettronica, Turin, Italy). On the VL, the electrode array was positioned on the distal third of the muscle belly, longitudinally to the muscle fibers. On the RF, the electrode array was positioned in the proximal third of the muscle belly along the longitudinal axis of the muscle. The reference electrode (24 mm, model: CDE‐S. OT Bioelettronica, Turin, Italy) was placed on the patella of the same limb. Before the placement of the electrodes, the skin area was shaved and then slightly abraded with abrasive paste and cleaned with water [32]. To ensure proper electrode–skin contact, the electrode cavities of the matrix were filled with conductive paste (Spes‐Medica, Battipaglia, Italy). The electrode arrays were fixed with an extensible tape.

### 2.4 Data Analysis

The torque, position, and EMG signals were amplified (gain 150), sampled at 2048 Hz, and converted to digital data with a 16‐bit A/D converter (Quattrocento; OT Bioelettronica, Turin, Italy). Signals, in single‐differential configuration, were visualized during acquisition and then stored on a personal computer using OT BioLab+ software version 1.5.5.0 (OT Bioelettronica, Turin, Italy) for further analysis.

#### 2.4.1 Torque Signals

Force values from the strain gauge were converted into torques using the individual distance from the rotating axis to the strain gauge. The torque signals were low‐pass‐filtered at 100 Hz using a fourth‐order zero‐lag Butterworth filter. MVT was defined as the highest torque calculated over an averaged window of 250 ms among the two maximal voluntary contractions. The onset of each contraction was visually assessed [33] by the same researcher through a hand‐customized MATLAB code. In the case of countermovement, the contraction was discarded. Torque was assessed at 50, 100, and 150 ms after the contraction's onset (RTD_50_, RTD_100_, RTD_150_). RTD_peak_ was calculated as the maximum of the first derivative of torque over time adopting a moving average of 20 ms. In isometric contractions, of 10 ballistic contractions, we averaged the torque and EMG variables (see next paragraph) calculated from the three best contractions, defined as those with the highest RTD_peak_, to increase the precision of the estimates.

To determine the individual torque–velocity relationship, torque and velocity were averaged from 80° (starting position) to 140° knee angles since this range of motion was covered by all participants in every load, without any active deceleration at the end of the contraction. A curvilinear function, derived from Hill's function [34], was used to fit averaged torque and velocity data to obtain torque–velocity relationships:

(1) $$T\left(V\right)=-T_{0}\frac{V-V_{0}}{V_{0}+\mathrm{CV}}$$

with *T* and *V* the averaged torque and velocity over the movement, *T*_0_ and *V*_0_ the torque and velocity–axis intercepts, and *C* the curvature index of the torque–velocity relationship.

**FIGURE 4:**
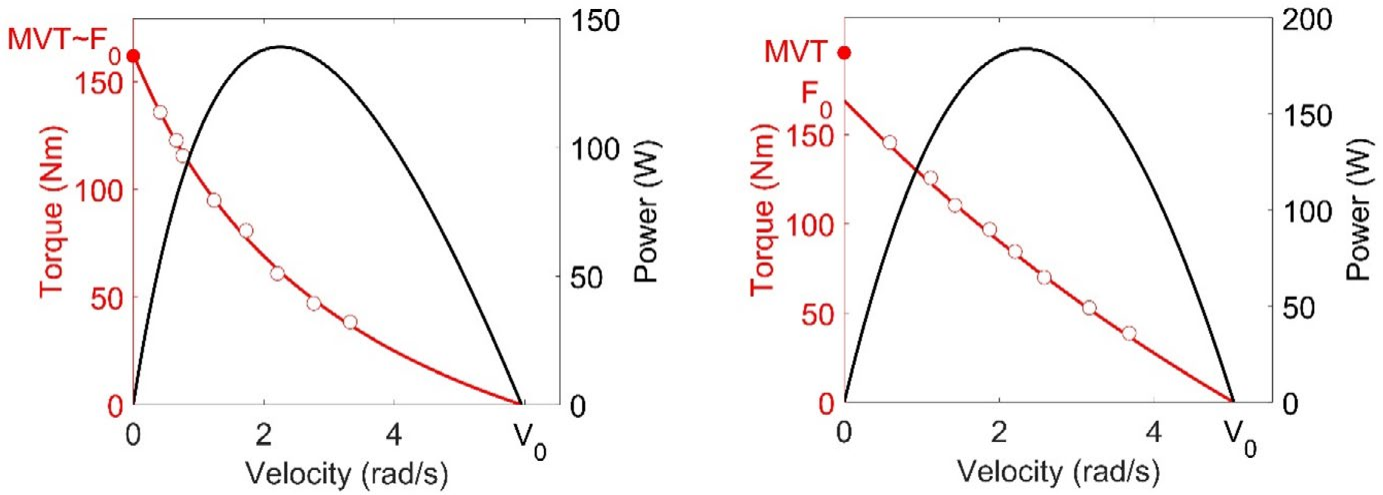
Torque–velocity and power–velocity relationship calculated over a leg extension nonisokinetic exercise in two male participants. The isometric maximal voluntary torque (MVT), maximal theoretical velocity (*T*_0_), and maximal theoretical velocity (*V*_0_) are also reported. On the left is reported a participant with a curvature *C* of the torque–velocity of 1.68 (*R* ^2^ = 0.998), while on the right is reported a participant with a more representative *C*, compared to the average of this study, of 0.31 (*R* ^2^ = 0.999).

Power–velocity relationships were then derived from torque–velocity relationships. The data extracted from the torque–velocity and power–velocity relationships were thus: C, *T*_0_, *V*_0_, *P*_max_ (apex of the power–velocity relationship), velocity at *P*_max_ (V_opt_), and torque at *P*_max_ (T_opt_). Representative examples of torque–velocity and power–velocity relationships are reported in Figure 4.

#### 2.4.2 High‐Density Surface Electromyography

The EMG signals were band‐pass‐filtered at 30–450 Hz using a fourth‐order zero‐lag Butterworth filter prior to analysis. We first removed the EMG channels showing excessive noise or artifacts through visual analysis. Signals for each column of electrodes were visually inspected, and the three to eight (depending on columns) single‐differential HDsEMG signal channels with clear action potential propagation without shape change were chosen for the analysis. In general, on the RF, no propagations of action potential could be seen (see Figure 2). The EMG onset is arbitrarily set at 20 ms before the torque onset, in accordance with previous studies of the quadriceps during voluntary contraction. This does not influence the EMG outcomes, as it aligns with the typical electromechanical delay observed in this muscle group. The amplitude of voluntary HD‐sEMG signals was assessed as the root mean square (RMS) across all available channels. RMS during maximal voluntary contractions was calculated in the same 250 ms epoch as MVT. RMS in ballistic contractions was calculated over 50, 100, and 150 ms from EMG onset. Then, RMS was normalized to the RMS calculated during maximal voluntary contractions and subsequently averaged across channels to obtain a single value for each muscle (e.g., for VL: RMS_50_VL, RMS_100_VL, RMS_150_VL). Moreover, RMS was also averaged across VL and RF to obtain a single estimate calculated over the first 50, 100, and 150 ms from EMG onset (RMS_50_VL + RF, RMS_100_VL + RF, RMS_150_VL + RF) [25].

#### 2.4.3 Statistical Analysis

Descriptive statistics are presented as mean ± SD and coefficient of variation (CV, %). Statistical analyses were performed using JASP (version 0.18) and statistical significance was set at *p* < 0.05. The relationship between individual predictor variables calculated under isometric conditions (RTD and EMG variables, independent variables) and the parameters calculated on the torque– and power–velocity relationships (dependent variables) was assessed through multiple stepwise linear regressions. In addition to stepwise regressions, Pearson's correlations were tested between the different variables to have a general view of the direct associations between isometric and dynamic indices and help to interpret results.

## 3 Results

### 3.1 Torque–Velocity Relationship and Interindividual Variability

Individual torque–velocity relationships were well fitted by the Hill's function (Equation (1)) with median *R* ^2^ of 0.991 (individual *R* ^2^ from 0.961 to 1.00). The range covered by experimental points was in average from 12% ± 5% to 70% ± 9% of *V*_0_ (Figure 3). The descriptive statistics and CV of the variables of interest are reported in Table 1. The MVT of knee extensors was 217 ± 44 Nm, with a CV of 28.6%. The peak torque reached during the isometric ballistic contractions was 86.6% ± 6.6% of MVT. The CV of selected variables ranged from 9% to 43%, demonstrating the large heterogeneity of participants' characteristics (Table 1). On average, the EMG and torque parameters calculated in the early phase of contraction showed larger intersubject variability compared to those calculated at later intervals. Indeed, the CV was ≈40% at 50 ms and ≈30%–20% at 100 ms and at 150 ms, respectively.

**TABLE 1:** Descriptive statistics of the parameters extracted from isometric ballistic contractions and from the torque–velocity relationship.

Isometric maximal torque (MVT) and rate of torque development (RTD)										
	MVT (Nm)	Time to RTDpeak (s)	RTDpeak (Nm/s)	RTDpeakN (MVT/s)	RTD50 (Nm/s)	RTD100 (Nm/s)	RTD150 (Nm/s)	RTD50N (MVT/s)	RTD100N (MVT/s)	RTD150N (MVT/s)
Mean	181.9	0.047	2210.9	12.5	722.4	941.6	848.1	4.1	5.3	4.7
SD	51.9	0.009	788.3	4.6	345.5	281.5	229.6	1.7	0.8	0.5
CV	0.286	0.201	0.357	0.368	0.478	0.299	0.271	0.427	0.161	0.100
Root mean square (RMS) of electromyographic signal in isometric ballistic contractions, normalized to maximal voluntary contraction										
	RMS_50_VL	RMS_50_RF	RMS_100_VL	RMS_100_RF	RMS_150_VL	RMS_150_RF	RMS_50_VL + RF	RMS_100_VL + RF	RMS_150_VL + RF	
Mean	69.4	69.0	89.8	86.6	92.7	91.6	69.2	88.2	92.1	
SD	28.8	27.1	24.1	25.0	21.0	23.5	25.0	22.6	20.1	
CV	0.414	0.393	0.268	0.288	0.227	0.256	0.361	0.257	0.218	
Parameters extracted from the torque velocity relationship										
	*V*_0_ (rad/s)	*T*_0_ (Nm)	C	*R* ^2^	*P*_max_ (W)	*T*_opt_ (Nm)	*V*_opt_ (rad/s)			
Mean	5.1	148.5	0.360	0.991	148.7	68.3	2.372	0.377		
SD	0.9	36.3	0.492	0.011	58.7	16.9		0.159		
CV	0.187	0.244	1.368	0.011	0.395	0.247				

### 3.2 Relationship Between Isometric RTD and TV Relationship Parameters

The correlations between *T*_0_ and *V*_0_ (*r* = 0.075) and between *T*_0_ and C (*r* = 0.213) were nonsignificant (*p* > 0.103). The correlation between *V*_0_ and C was low (*r* = 0.281, *p* = 0.029). Stepwise linear regressions showed that the main determinant of *V*_0_ was normalized RTD_50_ (*R* ^2^ = 0.145, standardized *β* = 0.380, *p* = 0.004); the main determinants of *T*_0_ were MVT (*R* ^2^ = 0.760, standardized *β* = 0.802, *p* < 0.001), followed by RTD_50_ (*R* ^2^ = 0.017, standardized *β* = 0.149, *p* = 0.046); the main determinant of *P*_max_ was RTD_150_ (*R* ^2^ = 0.612, standardized *β* = 0.782, *p* < 0.001); the main determinant of optimal velocity was normalized RTD_100_ (*R* ^2^ = 0.131, standardized *β* = 0.363, *p* = 0.006); the main determinant of optimal torque was MVT (*R* ^2^ = 0.757, standardized *β* = 0.878, *p* < 0.001). *C* was not significantly determined by any RTD parameter (Table 2).

**TABLE 2:** Results of the linear regression analysis.

	Outcomes: Torque‐velocity relationship parameters					
	*V*_0_	*T*_0_	*P*_max_	*T*_opt_	*V*_opt_	*C*
Determinants	RTD50 *R* ^2^ = 0.145	MVT *R* ^2^ = 0.760	RTD150 *R* ^2^ = 0.612	MVT *R* ^2^ = 0.757	RTD100 *R* ^2^ = 0.131	~
		RTD50 *R* ^2^ = 0.017				

The Pearson's correlation coefficients with heat maps between predictor variables calculated on the isometric testing and the TVP parameters are reported in Figure 4. Briefly, *V*_0_ was mostly correlated with indices calculated over the first 50 ms (i.e., RTD_50_, normalized RTD_50_, and RMS_50_VL + RF), while *T*_0_ was mostly correlated with MVT and late phase RTD (i.e., RTD_100_ and RTD_150_). Similarly to *T*_0_, *P*_max_ and *T*_opt_ were mostly correlated with MVT and late phase RTD (i.e., RTD_100_ and RTD_150_). Similarly to *V*_0_, velocity at maximal power was mostly correlated with indices calculated over the first 50 ms (i.e., RTD_50_, normalized RTD_50_, and RMS_50_VL + RF).

## 4 Discussion

We aimed to establish the contribution of isometric RTD and MVT to dynamic force production capacities extracted from the torque–velocity relationship, that is, the theoretical maximal velocity (*V*_0_), torque (*T*_0_), and maximal power of the knee extensors (*P*_max_). The main findings were that *V*_0_ and *V*_opt_ were mostly correlated with indices calculated over the first 50 ms (i.e., RTD_50_, normalized RTD_50_, and RMS_50_), while *T*_0_, *P*_max_, and *T*_opt_ were mostly correlated with MVT and late phase RTD (i.e., RTD_100_ and RTD_150_).

The quality of the TV relationship showed a good fit with a median *R* ^2^ of 0.991. The TV parameters are consistent with those of previous studies assuming isoinertial loads. However, compared to the parameters extracted previously [3], we found lower *T*_0_ (148 vs. 226 Nm) and *V*_0_ (5 vs. 17 rad/s) because while we averaged the torque and velocity values from the starting angle to 140°, they removed the first 30° of ROM and averaged these values from 100° to 130° when the contraction started at 70° of knee angle. The difference in averaging between the two studies has more effect on velocity than on torque, as in our approach we included all the initial phase of movement where the velocity is particularly low. As the lightest load, they took the free movement of the leg extension without any additional load. For this reason, the experimental condition with the maximum velocity corresponds to a slightly higher velocity of *V*_0_, that is, 75%, compared to 70% in our study. The curvature of the TV relation was also affected by the difference in the averaging between the two studies, as it was greater at 0.96 (data extracted from their Figure 5 of Hauraix et al. [3]) compared to 0.36 in our study.

**FIGURE 5:**
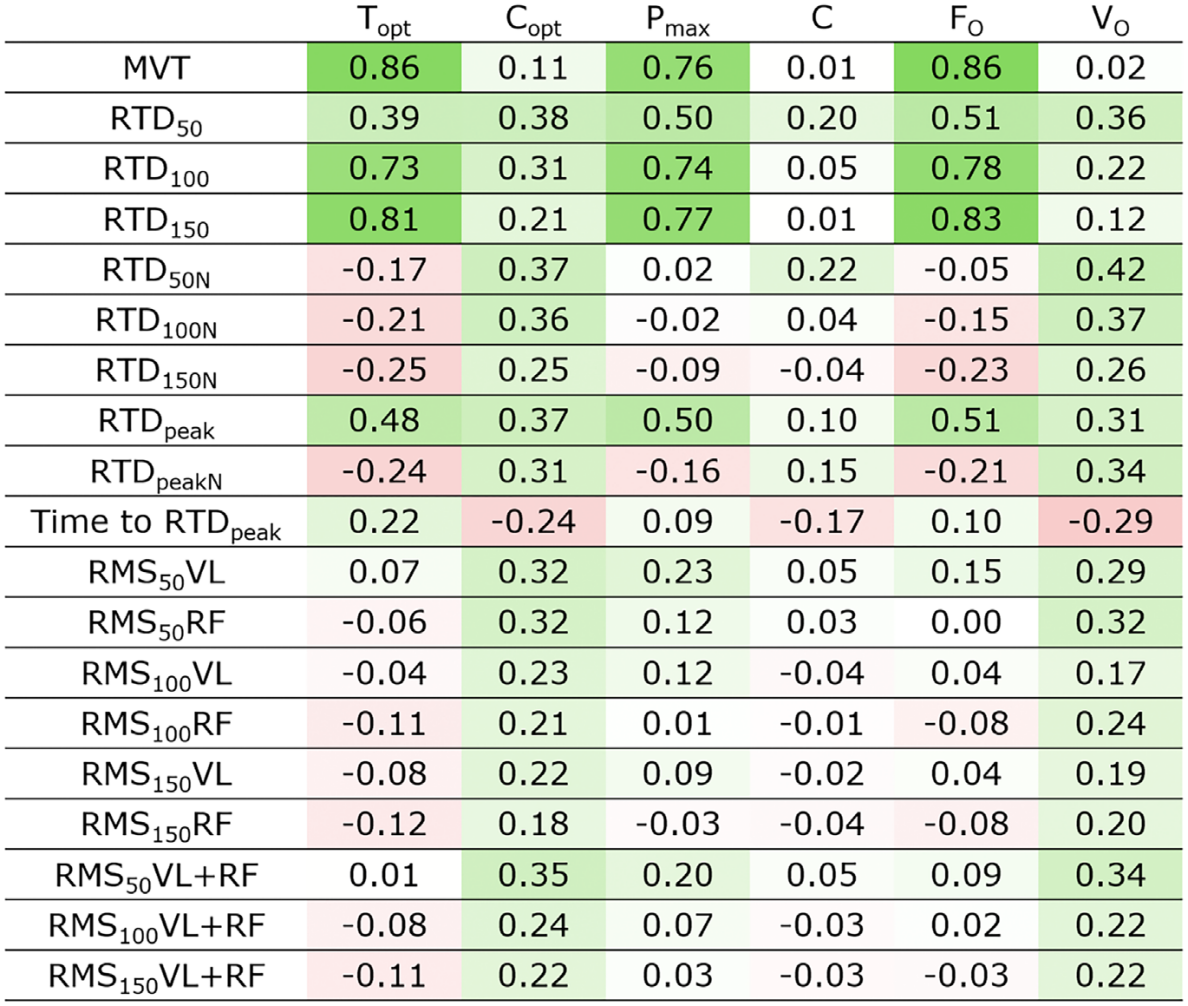
Pearson's correlation coefficients are shown between the determinants (in columns) calculated during isometric rapid contractions such as maximal voluntary torque (MVT), rate of torque development (RTD), electromyographic signal amplitude (root mean square, RMS) calculated over the first 50, 100, and 150 ms, and the parameters calculated from the torque–velocity relationship (in rows). Significant correlations are presented by *, **, and *** for *p* < 0.05, 0.01, and 0.001, respectively.

The most relevant finding of this study was that the capacity to activate muscles (RMS_50_) and produce torque (RTD_50_, normalized RTD_50_) at the beginning of a ballistic isometric contraction (i.e., in the first 50 ms) partly determines the capacity to produce torque at high velocity (*V*_0_). This was expected as, in high‐velocity movements, the time available to produce torque is short and thus being able to rapidly increase activation and consequently torque in the first instants is fundamental. The capability to increase muscle activation at the beginning of a contraction measured in the isometric test [23, 24, 25, 27, 28] is therefore important in tasks requiring high joint velocity. During fast concentric contractions, agonist muscle activation tends to be higher than in isometric conditions [35, 36]. Consequently, the muscle activation measurements that we obtained during isometric contractions may not directly correspond to those measured during dynamic conditions. The normalized RTD metrics demonstrated a stronger correlation with *V*_0_ than absolute RTD metrics (Figure 5). This suggests that the absolute torque capacities are not the primary factor, but rather the explosiveness, which can be defined as the capacity to rapidly produce torque at the net of maximal strength. The RMS of HD‐EMG signals calculated over RF and VL was more correlated with *V*_0_ than the RMS of individual muscles. This shows that the overall quadriceps activation can be better represented by taking into account both monoarticular and biarticular muscles, as they might have different activation profiles [37].

As expected, *T*_0_ was explained for 75% by MVT. Despite being well correlated, MVT and *T*_0_ do not represent the same force capacity. MVT is the maximal torque actually measured in isometric conditions, and so at a given knee angle, while *T*_0_ is the projection at null velocity of maximal torque measured under dynamic conditions over a range of motion. For this reason, MVT was on average ≈23% greater than *T*_0_ as it is known that isometric conditions are advantageous for producing maximal torque [38, 39]. *P*_max_, being the combination of *T*_0_ and *V*_0_, was mostly explained by RTD_150_. This underlies that the capacity to produce power is also determined by rapid torque production, but with the addition of the importance of maximal torque development since late‐phase non‐normalized RTD indices are known to be affected by MVT [23, 25]. This is in line with the high simple correlation between MVT and maximal power (*R* = 0.757). Therefore, the individual's maximal strength and the ability to increase quickly the torque are important capacities to produce maximal power.

For the curvature C of the TV relationship, no association with RTD or MVT was observed. For given *T*_0_ and *V*_0_, C represents the force production capacities at intermediate velocities. In other words, C can be interpreted as *P*_max_ normalized by *T*_0_ and *V*_0_. This means that the underlying mechanisms of the curvature of the TV relationship may affect *P*_max_ without influencing *T*_0_ and *V*_0_. The lack of association here between C and RTD or MVT would mean that the underlying mechanisms of RTD (e.g., the motor unit recruitment speed and firing rate or elastic structures stiffness [40]) or MVT (e.g., muscle cross‐sectional area or level of voluntary activation [41]) are different from those of C (e.g., the myosin cross bridges attachments and detachment rate [42]). If RTD or MVT can be expected to affect averaged force at intermediate velocities, they do not influence C values since they are also associated with *V*_0_ and *T*_0_, respectively. Although no correlation was expected between C and *T*_0_ or *V*_0_ (since C is conceptually independent from both *T*_0_ and *V*_0_), a correlation was observed between C and *V*_0_. This may be attributed to the fitting procedure since the experimental points in the torque–velocity relationship were closer to *T*_0_ than to *V*_0_. As a result, the estimation of *T*_0_ was likely more accurate, with a narrower confidence interval, while *V*_0_ was more extrapolated and likely more influenced by the estimation of the curvature parameter C, leading to the observed correlation between C and *V*_0_.

### 4.1 Methodological Choices and Limitations

The present results may depend on the range of motion chosen to characterize force production. The effect of RTD on dynamic force production and the TV relationship could vary if the angle range over which force and velocity are averaged is different, or if the TV relationship is based on peak values. However, considering average values, as we did in this study, is more relevant in characterizing functional abilities [12]. This is because the capacity to move a part of or the entire body depends on the mechanical impulse or work done over the entire movement, and so on the averaged torque exerted during the whole movement, not on the peak torque only. Similarly, the use of a nonisokinetic modality is a key strength of our study, as it is more functional and better reflects the force production capacities used in sports activities or daily life tasks, compared to isokinetic methods. Moreover, these modalities make it possible to reach higher velocities values [3]. We acknowledge that assessing muscle contractile function through evoked contractions and analyzing muscle fiber composition would have enhanced the interpretation of our results. Finally, although participants were given the opportunity to familiarize themselves with both isometric and dynamic tasks, the inclusion of a dedicated familiarization session could have increased their confidence in performing the required contractions.

### 4.2 Perspective

In sports and movements that require high shortening velocities, it is important to focus on the ability to generate torque in the first instant of contraction. This is because the limited time available to produce torque means that only a fraction of the maximum torque can be exerted. It should be noted that only a fraction of the theoretical maximum velocity can be attributed to the capacity to produce torque in the first few moments of contraction. This means that the other physiological determinants of maximal theoretical velocity such as muscle fiber composition [2], fascicle shortening velocities [3], and muscle belly gearing [43] should be investigated and described in light of this findings.

## 5 Conclusion

The capacity to voluntarily activate muscles and increase force in the first 50 ms of a ballistic contraction partially determines the capacity of producing torque at high velocity over a complete range of motion. Conversely, maximal isometric strength and late‐phase RTD (i.e., 150 ms after the onset of a contraction) are more relevant for producing maximal force and power. Therefore, when dynamic force production capacities are considered over a complete movement, the capacity to produce torque at high velocity partly depends on the capacity to raise the torque quickly in the early phase of the contraction, suggesting that some underlying determinants of RTD would also affect *V*_0_, but not the curvature of the TV relationship.

## Author Contributions

Gennaro Boccia, Paolo Riccardo Brustio, and Pierre Samozino conceived the study. Francesco Salvaggio, Arianna Pintore, Ludovico Grossio, and Elena Calcagno collected the data. Gennaro Boccia and Paolo Riccardo Brustio supervised the data collection. Gennaro Boccia, Francesco Salvaggio, and Ludovico Grossio analyzed the data. Gennaro Boccia performed the statistical analysis. Gennaro Boccia and Pierre Samozino wrote the draft, and Alberto Rainoldi, Paolo Riccardo Brustio, and Ludovico Grossio revised the draft.

## Conflicts of Interest

The authors declare no conflicts of interest.

## Data Availability

The data that support the findings of this study are available from the corresponding author upon reasonable request.
